# Caring ability of family caregivers of patients on cancer treatment: associated factors*

**DOI:** 10.1590/1518-8345.2824.3048

**Published:** 2018-10-11

**Authors:** Larissa de Carli Coppetti, Nara Marilene Oliveira Girardon-Perlini, Rafaela Andolhe, Maria Gaby Rivero de Gutiérrez, Steffani Nikoli Dapper, Fernanda Duarte Siqueira

**Affiliations:** 1 Universidade Federal de Santa Maria, Programa de Pós-Graduação em Enfermagem, Santa Maria, RS, Brazil.; 2 Universidade Federal de São Paulo, Escola Paulista de Enfermagem, São Paulo, SP, Brazil.; 3 Universidade Federal de Santa Maria, Programa de Pós-Graduação em Administração, Santa Maria, RS, Brazil.; 4 Faculdade Integrada de Santa Maria, Departamento de Administração e Gestão Comercial, Santa Maria, RS, Brazil.

**Keywords:** Caregivers, Home Nursing, Chronic Disease, Neoplasms, Nursing, Cross-Sectional Studies

## Abstract

**Objective:**

To analyze the association between the caring ability of family caregivers of patients on cancer treatment with the demographic and clinical characteristics of the patients, as well as the sociodemographic characteristics of the caregivers and of the care provided.

**Methods:**

A cross-sectional study was conducted with 132 family caregivers of patients on cancer treatment whose data were collected through the Brazilian version of the Caring Ability Inventory and questionnaires characterizing patients, caregivers and the care provided. Student’s t, Mann-Whitney or Kruskal-Wallis tests were used at the significance level ≤5%.

**Results:**

Patients’ age was significantly associated with overall caring ability (p = 0.002) and the caregiver’s dimensions courage (p = 0.006) and patience (p = 0.009). Caregivers’ education was associated with overall caring ability (p = 0.028) and the dimensions courage (p = 0.008) and patience (p = 0.045). Marital status was associated with the overall caring ability (p = 0.020); and the patience dimension (p = 0.045) and the time providing care with the patience dimension (p = 0.027).

**Conclusion:**

Caregivers of elderly patients who have higher schooling and do not have a partner demonstrate greater caring ability.

## Introduction

The Brazilian health situation is characterized by a process of accelerated epidemiological transition related to the fall of acute conditions and relative increase of chronic conditions^1^. Cancer, which is the second cause of death associated with chronic non-communicable diseases (CNCDs), is currently one of the most complex public health problems, given its epidemiological, social and economic magnitude^2^.

In addition to the high mortality rate, the consequences of CNCDs in Brazil and in the world have caused loss of quality of life and economic impact for families, the community and society due to the high degree of sequelae and disabilities that can affect the sick individuals^2^.

The process of illness, accompanied by dependency, demands adjustments in the family routine and the need for a situational transition, especially with regard to caring for the sick person. This caring is usually attributed to one of the family members, the caregiver^3^ ^-^ ^4^, who assumes responsibility for care at home mediated by the love and affection relationships that bind him or her to the dependent person, However, they may be or not prepared and able to assume such attribution^5^. 

In view of this, the responsibility and the commitment of care, when embraced by a person without preparation and previous orientations, may compromise the quality of care to be provided and bring physical and emotional changes to the caregivers themselves. Thus, support and education actions fostered by nursing professionals may favor the development or improvement of the ability to care. Such ability is understood as the potential of the person who assumes the role of caregiver of a significant family member or person who is disabled. Within this perspective, we include cognitive, instrumental and attitudinal dimensions that can be identified and measured according to indicators of knowledge, courage and patience^6^.

Considering that articles on the translation and validation of the instrument used to evaluate the care ability, the Caring Ability Inventory (CAI)^7^ into Brazilian Portuguese are recent and that the scientific production on this subject in our country is incipient, it was necessary to know the international researches evaluating this theme and using this instrument. To do so, we searched the bases Scielo, Lilacs, Ibecs, Medline, Pubmed and Scopus, using the terms “Caring Ability Inventory” OR “*Inventário de Habilidades de Cuidado*” OR “*Inventario de Habilidad de Cuidado*” in the field Words. When analyzing the objectives and results of the studies found, we verified that the instrument has been used to measure the care ability of several populations that perform the care activity, such as health professionals, nursing students and caregivers. However, most studies have approached the caregivers, demonstrating the professionals’ concern in knowing the abilities of these individuals to meet the demands of home care. 

Faced with the evidence, we could see in the studies that the factors associated with the caring ability arise from cultural, political and social contexts that are different from those of our country, since the researches were mostly conducted outside Brazil. Thus, there is a gap in the Brazilian knowledge and the need to develop national investigations that evaluate the care ability of family caregivers, considering that these results may serve as a subsidy for nursing care.

In this sense, a question arises: is there an association between the care ability and the characteristics of patients on cancer treatment, of family caregivers and of the care provided? The study hypothesis was that the caregivers’ ability to care for their family is associated with the demographic and clinical characteristics of patients undergoing oncological treatment, with the sociodemographic characteristics of the caregivers themselves and with the care they provide. Thus, the objective of the present study was to analyze the association between and the care ability of family caregivers of patients on cancer treatment and the patients’ demographic and clinical characteristics, the caregivers’ sociodemographic characteristics and the care provided.

## Methods

This is a cross-sectional study carried out between March and August 2017 with family caregivers of patients undergoing cancer treatment selected in the chemotherapy and radiation therapy sectors of a university hospital in the central region of Rio Grande do Sul.

The selection of caregivers began by the recognition of dependent patients being attended in the aforementioned sectors. Inclusion criteria were being the primary caregiver of a patient undergoing oncologic treatment with some degree of dependence on daily life activities; being aged 18 years or older; providing home care to the dependent family member. The exclusion criterion was presenting cognitive difficulties evident in the initial approach. 

The primary caregiver was defined as the family member with total or partial responsibility of care for the sick individual. In situations where there was more than one caregiver and they mentioned dividing care tasks the patient was asked who he/she considered his/her main caregiver, and this indication was respected.

The sample selection was non-probabilistic and by convenience. Thus, 160 dependent patients had been attended in chemotherapy and radiation therapy during the period of data collection. When the caregivers of these patients were contacted, 23 were not the primary caregivers, five showed communication and/or comprehension difficulties, two were under 18 years of age and five did not agree to participate in the study. Thus, the sample consisted of 132 caregivers, representing 82.5% of the population of dependent patients on cancer treatment in the period defined for the research. 

For data collection we used a questionnaire for the demographic and clinical characterization of the patients, a questionnaire for the sociodemographic characterization of the family caregivers and characteristics of the care provided and the Portuguese version of the Caring Ability Inventory (CAI-BR). The original name for the Inventory and the acronym “CAI-BR” in English remained the same for the Brazilian version, according to the posture of the authors responsible for the translation and cross-cultural adaptation of the instrument^7^.

The demographic and clinical variables of the patients were age, sex, medical diagnosis, diagnosis time and dependence for activities of daily living, verified by the Barthel Index, which aims to measure the degree of assistance required for activities of daily living, ranging from “0” (totally dependent) to “100” (independent)^8^. The medical diagnoses were organized according to the INCA classification of malignant tumors^9^. 

The sociodemographic characterization questionnaire of the family caregivers aimed at the characterization as to sex, age, schooling, marital status and family income; and the characteristics of the care provided aimed at the time of care, completion of training course for caregiver and previous experiences.

The CAI-BR aims to evaluate the abilities of an individual based on his/her own perception of his/her ability to provide care, taking into account instrumental and cognitive aspects. This scale comes from the Caring Ability Inventory (CAI)^6^, which was validated and translated into Spanish in 2005^10^ and into Brazilian Portuguese in 2016^7^. The inventory consists of 37 items divided into three dimensions: knowledge (understanding of self and others), courage (ability to face the unknown) and patience (tolerance and persistence), with 14, 13 and 10 items, respectively. Responses are organized in a Likert-type scale, ranging from 1 to 5, in which 1 means “I strongly disagree” and 5 “I strongly agree.” The total score and of each one of the dimensions of the instrument is obtained by the sum of the answers given to the items that compose it.

The mean (M) and the standard deviation (SD) were used to classify the scores into low, medium and high level of care ability, so that the SD interval below and above the mean was considered medium level. Below this value, it was considered low level and, above it, high level, according to the author of the original instrument^6^.

The reliability and validity of the original CAI were evaluated by Cronbach’s alpha and test-retest, which obtained the values of 0.84 and 0.80, respectively^6^. The Spanish version reports Cronbach’s Alpha of 0.86 and a Pearson Correlation Coefficient of 0.66^10^. In the Portuguese version, the Cronbach’s Alpha obtained a result of 0.78 and the Correlation Coefficient a score of 0.76^7^. In the present study, the internal consistency obtained for the total CAI-BR was 0.60. 

Data were typed in the Excel for Windows program (Office, 2011), and were stored and organized in a spreadsheet. After the verification and correction of typing inconsistencies, the data were analyzed electronically using the statistical software SPSS version 23.0. 

The qualitative variables were presented by the distribution of absolute and relative frequencies, and the quantitative variables, in measures of central tendency, SD and variation. Normality of the groups was tested using the Kolmogorov-Smirnov test. Student’s t-test was performed when both groups presented data with normal distribution, and the Mann-Whitney or Kruskal-Wallis test, when asymmetric. For all analyzes, the level of significance set was≤5%.

The study complied with the principles of Resolution No. 466/12 of the National Health Council and was submitted to the Research Ethics Committee (CEP) under Certificate of Presentation for Ethical Assessment (CAAE) no. 65195617.0.0000.5346 and approved under opinion no.1,977,316.

## Results

There was predominance of male patients (n = 78, 59.1%), aged between 29 and 91 years, mean age of 66.52 (SD = 12.69), with a diagnosis of digestive tract cancer (n = 32, 24.2%), followed by urological system cancer (n = 24, 18.2%) and head and neck cancer (n = 22, 16.7%). The time of diagnosis ranged from one month to 204 months, with an average of 24.09 (SD = 37.23) months. Regarding the degree of dependence, the score ranged from 10 to 90, showing a prevalence of moderate dependence (n = 95, 72.0%), followed by severe/total dependence (n = 37, 28.0%).

Regarding the characterization of the caregivers, there was a predominance of the female sex (n = 103; 78%), having a partner (n = 101; 76.5%) and mean age of 48.68 (SD = 14.01) ranging from 18 to 76 years. The schooling ranged from zero to 20 years, with an average of 9.08 years of schooling (SD = 4.61), which represents complete primary education. 

Family income ranged from 0.5 to 16.5 minimum wages, with a prevalence of two to three minimum wages (n = 36, 27.3%). The time of care was on average 10.18 months (SD = 14.79). None of the relatives had attended a caregiver training course; however, the majority had had previous experiences of care (n = 72, 54.5%), involving sick relatives and friends. 

Regarding the care ability, the total CAI-BR scores and its dimensions knowledge, courage and patience showed that the family caregivers presented medium level of ability, as shown in Table 1.

**Table 1:** Classification and scores of overall caring ability and its dimensions (n = 132). Rio Grande do Sul, Brazil, 2017

Variable	Mean (SD*)	Variation	Low	Medium	High
			n (%)	n (%)	n (%)
Knowledge	52.20 (3.54)	44-61	22 (16.67)	87 (65.91)	23 (17.42)
Courage	46.31 (3.98)	37-57	20 (15.15)	91 (68.94)	21 (15.91)
Patience	40.54 (2.71)	34-48	16 (12.12)	100 (75.76)	16 (12.12)
CAI-BR^†^ Total	139.07 (7.10)	125-160	22 (16.67)	89 (67.42)	21 (15.91)

*SD=Standard deviation; †CAI-BR = Caring Ability Inventory, Brazilian version

Table 2 shows that caregivers caring for male patients and aged over 66 years present higher levels of ability in the overall scale and in the knowledge and courage dimensions. In the patience dimension, caregivers of female patients older than 66 years showed higher scores. Regarding the degree of dependence, caregivers caring for patients in cancer treatment with severe/total dependence present a higher level of ability in the overall scale and in the courage and patience dimensions. In the knowledge dimension, the caregivers of patients with moderate degree of dependence obtained higher scores.

**Table 2:** Association of the demographic and clinical variables of the patients on oncological treatment and the family caregiver’s caring ability of (n=132). Rio Grande do Sul, Brazil, 2017

Patients’ variables	n (%)	Caring ability							
		Knowledge		Courage		Patience		Overall CAI-BR*	
		Mean(SD^†^)	*p-*value	Mean(SD^†^)	*p-*value	Mean(SD^†^)	*p-*value	Mean(SD^†^)	*p-*value
Sex									
Female	54 (40.9)	52.18(3.69)	0.975^‡^	46.05(4.37)	0.542^‡^	40.57(2.62)	0.899^‡^	138.81(7.05)	0.757^‡^
Male	78 (59.1)	52.20(3.35)		46.49(3.70)		40.51(2.77)		139.20(7.17)	
Age									
29-65 years old	57 (43.2)	51.74(3.81)	0.326^§^	45.33(4.00)	0.006^§^	39.93(2.90)	0.009^§^	137.00(7.47)	0.002^§^
66-91 years old	75 (56.8)	52.55(3.30)		47.05(3.82)		41.00(2.47)		140.60(6.43)	
Dependence									
Severe/total	37 (28.0)	52.16(4.01)	0.853^§^	46.94(3.35)	0.206^§^	40.62(2.61)	0.757^§^	139.73(7.06)	0.470^§^
Moderate	95 (72.0)	52.21(3.37)		46.06(4.19)		40.50(2.75)		138.78(7.13)	

*CAI-BR = Caring Ability Inventory, Brazilian version; †SD=Standard deviation; ‡Student’s t-test; §Mann-Whitney test

The associations expressed in Table 2 revealed a statistically significant relationship between the age of patients undergoing oncology treatment and the overall caring ability (p = 0.002), the courage (p = 0.006) and the patience (p = 0.009) of the family caregiver, demonstrating that caring for patients over the age of 66 requires from the caregiver a greater level of courage, patience and caring ability.

When analyzing the care ability and sociodemographic characteristics of family caregivers (Table 3), the male caregivers aged 58 to 67 years, with more than 16 years of schooling, with no partner, income greater than six minimum wages, having taken care of patients for 19 to 24 months and with previous experience obtained higher overall caring ability scores.

**Table 3:** Association of sociodemographic variables of family caregivers, characteristics of care provided and caring ability (n=132). Rio Grande do Sul, Brazil, 2017

Care and caregivers’ variables	n (%)	Caring ability							
		Knowledge		Courage		Patience		Overall CAI-BR*	
		Mean (SD^†^)	*p-*value	Mean (SD^†^)	*p-*value	Mean (SD^†^)	*p-*value	Mean (SD^†^)	*p-*value
Sex									
Female	103 (78.0)	52.39(3.54)	0.220^‡^	46.19(3.86)	0.528^‡^	40.44(2.74)	0.467^‡^	139.04(7.19)	0.984^‡^
Male	29 (22.0)	51.48(3.50)		46.72(4.39)		40.86(2.58)		139.07(6.87)	
Age									
18-37 years old	29 (22.0)	52.17(3.79)	0.922^§^	46.65(3.73)	0.900^§^	41.17(2.39)	0.321^§^	140.00(6.26)	0.492^§^
38-57 years old s	70 (53.0)	52.07(3.63)		46.24(4.30)		40.24(2.77)		138.56(7.43)	
58-76 years old	33 (25.0)	52.48(3.20)		46.15(3.53)		40.60(2.80)		139.24(7.18)	
Schooling									
0-4 years	22 (16.7)	52.23(3.45)	0.769^§^	45.54(2.70)	0.008^§^	39.45(1.79)	0.045^§^	137.22(5.50)	0.028^§^
5-8 years	45 (34.1)	51.82(3.75)		45.13(4.06)		40.37(3.01)		137.33(7.10)	
9 years or more	65 (49.2)	52.45(3.48)		47.38(4.03)		41.01(2.65)		140.85(7.21)	
Marital status									
With partner	101 (76.5)	51.95(3.31)	0.150^§^	46.02(3.79)	0.144^§^	40.27(2.68)	0.045^§^	138.26(6.87)	0.020^§^
Without partner	31 (23.5)	53.00(4.15)		47.22(4.46)		41.38(2.65)		141.61(7.33)	
Family income									
Up to 3 minimum wages	90 (68.2)	52.13(3.49)	0.781^§^	46.07(4.06)	0.190^§^	40.31(2.61)	0.477^§^	138.52(6.78)	0.334^§^
4 to 6 minimum wages^||^	37 (28.0)	52.26(3.66)		46.54(3.84)		40.91(2.79)		139.71(7.46)	
>6 minimum wages^||^	5 (3.8)	53.00(4.30)		49.00(2.74)		42.00(3.67)		144.00(9.53)	
Time of care									
1-3 months	47 (35.6)	51.55(3.35)	0.409^§^	45.85(3.56)	0.747^§^	39.76(2.16)	0.027^§^	137.17(5.78)	0.145^§^
4-6 months	36 (27.3)	52.92(3.56)		46.92(3.84)		40.67(3.10)		140.05(5.95)	
7-12 months	25 (18.9)	52.40(3.86)		45.96(4.78)		41.80(3.01)		140.16(9.79)	
>12 months	24 (18.2)	52.16(3.55)		46.67(4.13)		40.54(2.30)		139.37(7.36)	
Previous experience									
Yes	72 (54.5)	52.58(3.72)	0.171^‡^	46.27(3.62)	0.918^‡^	40.19(2.73)	0.110^‡^	139.06(6.60)	0.986^§^
No	60 (45.5)	51.73(3.28)		46.35(4.39)		40.95(2.63)		139.03(7.70)	

*CAI-BR = Caring Ability Inventory, Brazilian version; †SD= Standard deviation; ‡Student’s t-test; §Kruskal-Wallis test; || Minimum Salary Quotation in 01.01.2017 = R$937.00

In the knowledge dimension, the female caregivers aged between 48 and 57 years, with 11 to 15 years of schooling, with no partner, income greater than six minimum wages, having taken care of patients for more than 25 months and with previous experience were those who obtained the highest scores.

In the courage dimension, the male caregivers aged between 28 and 37 years, with more than 16 years of study, with no partner, income greater than six minimum wages, having taken care of patients for 19 to 24 months, and with no previous experience of care had achieved higher scores.

In the patience dimension, higher scores were observed among male caregivers aged 58 to 67 years, between 11 and 15 years of schooling, with no partner, income less than a minimum wage, having taken care of patients for seven to 12 months and no previous experience. 

In the analysis of the relationship between these variables (Table 3), it was found that schooling was associated in a statistically significant way with the overall caring ability (p = 0.028) and the dimensions courage (p = 0.008) and patience (p = 0.045). In the overall caring ability and in the courage dimension, a statistically significant difference was observed between the groups of caregivers with five to eight years and nine years or more years of schooling (p = 0.050, p = 0.009). In the patience dimension, this difference was between the groups with zero to four years and nine years or more (p = 0.042) of schooling. These results indicate that the higher the family caregiver’s level of schooling, the better his/her overall caring ability, as well as his/her courage and patience. 

When analyzing the marital status of the family caregivers, there was a statistically significant association in the patience dimension (p = 0.045) and in the overall ability (p = 0.020), showing that the caregivers with no partner presented higher levels of patience and overall ability.

Regarding the characteristics of care (Table 3), the patience dimension was associated in a statistically significant way with the time of care (p = 0.027). The difference was observed when comparing groups who have been taking care of patients for one to three months and those for seven to 12 months (p = 0.021), showing that the longer the time of care, the greater the family caregiver’s level of patience.

## Discussion

The rapid process of demographic transition observed in Brazil represents a challenge for public health, since as the age advances, there is a progressive development of CNCDs, considering that these affect the populations of greater age^1^. This perspective can be identified by considering the predominance of advanced age patients undergoing cancer treatment, as evidenced in this study and in similar results described in the literature^11^ ^-^ ^13^.

The Rio Grande do Sul state has the highest rates of human development, life expectancy and proportion of elderly people in relation to the general population. As a result, the likelihood of CNCDs increases, and in addition to the genetic components that predispose inhabitants to illness, the cultural and social factors of the region should be highlighted, such as inadequate nutrition, smoking, alcoholism and sedentary lifestyle, which may justify the findings of this study regarding the cases of digestive tract cancer^14^.

The moderate dependence prevailed among the patients, being associated with the need of aid for eating, hygiene, clothing, eliminations and locomotion. These findings are similar to those presented in an international study with elderly patients with chronic illness in home care, in which half of the participants had moderate dependence^15^. However, a national study of frail elderly living at home reveals that most of them is totally or severely dependent^16^. Both situations should be considered when analyzing together with the family of people on cancer treatment the conditions of care at home and the need for support for the primary caregiver.

Regarding family caregivers, the present study, like other studies with this population, identified that the majority was women, with a mean age of 48.68 years and with a partner^17^ ^-^ ^19^. This reinforces the social role of women still present in society, which comes from a historically determined construction and interconnected with the fact that women did not perform activities outside the home, which gave them greater availability - and learning possibility - for the care of the home, children and family^18^.

The low level of schooling evidenced among the participants of this study coincides with the literature, demonstrating the prevalence of family caregivers with less than four years of schooling or an average of five to eight years^16^ ^-^ ^18^. People with low schooling tend to dedicate themselves to household and other low-paid services, as society requires higher levels of education to enter in formal work^18^. Thus, low family income, an issue also observed in this research, may be related to this limitation as to the inclusion of the subject in the labor market and the consequent choice to move away from the work assignments to perform the care^20^, since hiring a caregiver is not feasible, especially in situations of prolonged dependence. 

In this sense, regarding the time spent for care, the caregivers of this research have been dedicated to this activity for ten months, on average. On the other hand, considering previous studies, the care time of the majority of caregivers exceeds three years, some of whom have been caring for more than ten years. Such evidence allows us to conclude that home care can last for days, months, years or even decades, because despite the technological advances in cancer treatment, the direct and permanent presence of the caregiver will be necessary as long as the person’s inability requires it^5^. On the other hand, the technological advance for the treatment of cancer can lead to sequelae or complications that require continuous care and follow-up.

The assumption of the role of family caregiver and the permanence in this function for a long period are presented in the present study as a tendency for caregivers, since once a caregiver, possibly always a caregiver^17^. This assumption may be related to the fact that the majority of the caregivers covered by this study had previous experiences of care in the family nucleus or with sick friends. The fact of having cared for someone in the past is a characteristic of the caregiver described in the literature^17^.

Regarding the evaluation of the caregiver’s ability to take care of the family members of this study, it was evidenced that most of them obtained score compatible with the medium level of ability, in the overall CAI-BR, as well as in their dimensions, namely knowledge, courage and patience. These results partially diverge from those described in international studies, which showed that most caregivers presented a high level of ability in the overall score, as well as in the knowledge and patience dimensions. In the courage dimension, the result is similar to that of this study, since most of the caregivers presented medium level of ability^5^ ^,^ ^21^. The identified differences can be related to the health education and support actions offered to the caregivers by the services where the data were collected, which are focused on the preparation of the families and the improvement of the caring ability. Therefore, it is important to emphasize the importance and need of the health services to establish structured counseling programs for the families of people undergoing cancer treatment in order to support them in their responsibility to continue providing the care their relatives need.

The age of the dependent patient was associated with the level of the family caregiver’s overall ability, courage and patience, which allows us to infer that patients with advanced age require higher levels of courage, patience and ability on the part of the caregiver to perform the care. The elderly, previously healthy and independent, when faced with the process of illness, can perceive in a negative way the conditions that limit their independence, as well as the need to have another person’s help to be able to develop their daily activities. Thus, the family caregiver needs not only to be courageous - understood as the ability to face the unknown that the other and the situation represent - and to strive to respond to the elderly patient’s demands, but also to demonstrate patience, tolerance and persistence, giving time and space for the individual to adapt to the circumstances at their own pace and in their own way. 

From this perspective, the educational level and socioeconomic status may be a positive influence so that the caregiver has higher levels of caring ability, however, the results of this investigation show that the level of education is statistically significant with the dimensions courage, patience and overall caring ability, demonstrating that caregivers with a higher educational level have greater caring ability, courage and patience. This may be justified by the fact that these individuals have more conditions to access and search information in face of emerging care requirements, thus favoring the practice and development of the caring ability. 

Another factor that to be considered when assessing the caring ability is the family caregiver’s marital status because the findings of this study show that not having a partner positively influences levels of patience and overall ability. This evidence diverges from the literature, which mentions that living with a partner can provide more ability, especially in the dimensions patience and knowledge, because it allows the person to relate to and to live with the other^22^. On the other hand, not having a partner may represent more time for care and greater patience, since the caregiver does not become involved with the demands inherent to the conjugal situation.

Patience can also be related to time, that is, the time spent on the caring activity. This was verified in this study that presented a statistically significant relationship, demonstrating that patience increases over the time of care. This finding is similar to the literature, in which it was observed that care time of less than six months is related to lower levels of patience, which may be compatible with effort and fatigue resulting from the process of adaptation to the new situation of care to the dependent family member^23^.

Caregivers who had more than 25 months of care and previous experiences in care showed higher scores in the knowledge dimension. Although this result was not statistically significant, it demonstrated evidence that caregiving time and experiences are a way of learning, since they allows the knowledge about the other to be constructed, enabling to understand who is the person cared for, what their needs, strengths and weaknesses are and what strengthens their well-being. In addition, it favors the knowledge of one’s own capacities and limitations.

This study presents important data on who the dependent patients on cancer treatment are, on the family caregivers and on how they provide care at home, which represents a contribution to the nursing practice and to the construction of interventions that aim at strengthening, qualifying and developing attributes that are fragile in their caring ability in terms of knowledge, courage, and patience. Also, it allows knowing which factors are associated with the caring ability, thus enabling to meet the individual and emergent needs of the family caregiver and of the care practice beyond the hospital environment. 

The limitations of this study are related to the bias of temporality, because it is a cross-sectional study, as well as to the access to the participants, because it is restricted to a public service specialized in oncology, which may compromise the generalization of results. 

It is also worth noting that the study developed with the purpose of culturally adapting the CAI-BR was a pioneer in the Brazilian scenario in search of an instrument that measures the construct “caring ability”, having as participants informal caregivers. However, there is a lack of research at the national level that addresses this issue, evidencing the need to develop studies that, in addition to improving the instrument used, increase the knowledge of this reality in the country and compare it with that developed in other countries in order to obtain knowledge that could support the planning of public policies and institutional actions aimed at the support of caregivers of people with chronic illness.

## Conclusions 

The results of the present study confirmed the hypothesis that family caregivers’ caring ability is associated with the demographic and clinical characteristics of patients undergoing oncologic treatment, with the sociodemographic characteristics of the caregivers themselves and with the care they provide. 

Statistically significant associations between the age of the cancer patient and the degree of schooling of the family caregiver with the overall caring ability and the courage and patience dimensions, widely recognized as promoters of quality of care, constitute some of the evidence found in this investigation. On the other hand, the significant association between the family caregiver’s marital status and overall caring ability and patience dimension demonstrates the multifactorial nature of the aspects related to the caring ability.

Moreover, the association between time of care and the patience dimension, as observed in this research, brings light to the approach to those who take the care demands and can be an important tool when planning health education actions provided to these people.

Thus, the nurse, as an educator par excellence, has the potential to manage, plan and carry out joint practices with family caregivers, valuing them as partners in the process of caring for the patient on cancer treatment.
